# 2024 Update on Position Statement by Experts from the Polish Society of Allergology and the Polish Respiratory Society on the Evaluation of Efficacy and Effectiveness of Single Inhaler Triple Therapies in Asthma Treatment

**DOI:** 10.3390/arm92060041

**Published:** 2024-10-31

**Authors:** Paweł Śliwiński, Adam Antczak, Adam Barczyk, Adam J. Białas, Małgorzata Czajkowska-Malinowska, Karina Jahnz-Różyk, Marek Kulus, Piotr Kuna, Maciej Kupczyk

**Affiliations:** 1 2nd Department of Respiratory Medicine, Institute of Tuberculosis and Lung Diseases, 01-138 Warsaw, Poland; 2 Department of General and Oncological Pulmonology, Medical University of Lodz, 90-419 Lodz, Poland; adam.antczak@umed.lodz.pl; 3 Department of Pneumology, Faculty of Medical Sciences, Medical University of Silesia, 40-635 Katowice, Poland; adagne@icloud.com; 4 Department of Pneumonology, Medical University of Lodz, 90-419 Lodz, Poland; adam.bialas@umed.lodz.pl; 5 Department of Pulmonary Rehabilitation, Regional Medical Center for Lung Diseases and Rehabilitation, Blessed Rafal Chylinski Memorial Hospital for Lung Diseases, 91-520 Lodz, Poland; 6 Department of Lung Diseases and Respiratory Failure, Kuyavian-Pomeranian Pulmonology Center, 85-326 Bydgoszcz, Poland; m.cz.malinowska@interia.pl; 7 Department of Internal Medicine, Pneumonology, Allergology and Clinical Immunology, Military Medical Institute–National Research Institute, 04-141 Warsaw, Poland; krozyk@poczta.onet.pl; 8 Department of Pediatric Pneumonology and Allergology, Warsaw Medical University, 02-091 Warsaw, Poland; marek.kulus@wum.edu.pl; 9 Department of Internal Medicine, Asthma and Allergy, Medical University of Lodz, 90-153 Lodz, Poland; piotr.kuna@barlicki.pl (P.K.); maciej.kupczyk@umed.lodz.pl (M.K.)

**Keywords:** asthma, asthma treatment, ICS/LABA, LAMA, single inhaler triple therapy, SITT

## Abstract

**Highlights:**

**What are the main findings?**

- Inhaled triple-combination medications, ICS/LABA/LAMA, within a singular inhalation device yield a dual benefit of minimizing the likelihood of adverse effects linked with the principal therapeutic agent for asthma management, namely ICS, while concurrently optimizing bronchodilator efficacy.
- SITT therapy has the potential to enhance asthma symptom management and postpone the requirement for initiating biologic therapy.

**What are the implications of the main findings?**

- SITT therapy is proposed as a first-line strategy over other alternative methods for intensifying asthma treatment.

**Abstract:**

Medication non-adherence remains a substantial obstacle in asthma care, prompting the exploration of novel therapeutic modalities that prioritize rapid symptom relief, anti-inflammatory activity, and facilitate patients’ compliance. This task is well-served by the following new form of therapy: inhaled triple-combination medications ICS/LABA/LAMA (inhaled glucocorticosteroid/long-acting beta2-agonist/long-acting muscarinic antagonist). The integration of three medications within a singular inhalation device culminates in the reduction of the effective dose of the principal therapeutic agent for asthma management, namely ICS. This consolidation yields a dual benefit of minimizing the likelihood of adverse effects typically linked with ICS while concurrently optimizing bronchodilator efficacy. The accumulated evidence suggests that adding LAMA to a medium- or high-dose ICS/LABA results in a decrease of asthma exacerbations compared to medium- or high-dose ICS/LABA alone, accompanied by sustained enhancements in lung function parameters. In adult patients experiencing suboptimal asthma control despite medium/high-dose ICS/LABA treatment—regardless of adherence to GINA-recommended strategies, such as MART therapy as a first-line approach, or alternative second-line strategies—we propose that the preferred course for intensifying asthma therapy involves the addition of a LAMA, ideally in the form of SITT.

## 1. Introduction

Asthma represents a substantial clinical burden, affecting a significant segment of the global population. According to data from the National Health Fund (NHF) [1], the prevalence of asthma in Poland stood at 1.97 million individuals in 2022. This calculation, derived from payer records, indicates a nearly 10% reduction compared to estimates from 2019. The number of patients receiving medical attention with a primary diagnosis of asthma in 2022 was 1.02 million, marking an 8% decline from 2019. At the same time, 2.9 million patients obtained prescriptions for reimbursed asthma medications in 2022, representing the highest observed figure since 2013. The multifaceted nature of this trend necessitates an ongoing investigation into its underlying determinants.

Despite the widespread availability of inhalation therapies for asthma management, the percentage of patients filling prescriptions for inhaled glucocorticosteroid (ICS), for example, remains suboptimal. The NHF report underscores this issue, revealing that within the 18–40 age bracket, only 14.7% of patients obtained prescriptions for reimbursable medications containing budesonide, with a defined daily dose (DDD) sufficient to cover at least 33.3% of the theoretical demand for this agent. Corresponding figures for the 41–55 and 56–70 age cohorts were 30.7% and 42%, respectively.

Adherence to therapeutic recommendations in respiratory diseases remains notably deficient, presenting a persistent challenge in asthma management [2]. Estimates suggest that 30%–70% of asthma patients fail to comply with prescribed treatments [3,4], while up to 50% exhibit improper inhaler technique despite adequate training [5].

Consequently, medication non-adherence remains a substantial obstacle in asthma care, prompting the exploration of novel therapeutic modalities that prioritize rapid symptom relief, particularly dyspnea. This task is well-served by combination medications containing two active ingredients, ICS/LABA (inhaled glucocorticosteroid/long-acting beta2-agonist) and a new form of therapy, that being inhaled triple-combination medications ICS/LABA/LAMA (inhaled glucocorticosteroid/long-acting beta2-agonist/long-acting muscarinic antagonist) [6].

A strategy for the use of registered single inhaler triple therapy (SITT) is outlined in the Global Initiative for Asthma (GINA) report [7]. The novelty of this inhaled therapy is the addition of a second bronchodilator from the cholinolytic group to the primary medications for the treatment of asthma, which include an anti-inflammatory glucocorticosteroid and a beta-2-agonist bronchodilator.

The integration of three medications within a singular inhalation device culminates in the reduction of the effective dose of the principal therapeutic agent for asthma management, namely inhaled glucocorticosteroids. This consolidation yields a dual benefit of minimizing the likelihood of adverse effects typically linked with ICS, while concurrently optimizing bronchodilator efficacy [8].

## 2. Recommendations to Date

In June 2021, the Expert Group of the Polish Society of Allergology and the Polish Respiratory Society formulated a position statement concerning newly registered inhaled triple-combination therapies tailored for the management of asthma within Poland.

At that time, clinical situations were identified in which patients might benefit most from the addition of cholinolytics (LAMAs) to an already ongoing therapy consisting of inhaled glucocorticosteroids (ICSs) and beta2-agonists (LABAs). The efficacy of these treatment modalities was also determined based on the results of clinical trials, with the aforementioned substances administered from a single inhaler triple therapy (SITT) [9].

The findings derived from randomized clinical trials (RCTs) investigating the efficacy of the newly registered single inhaler triple therapy (SITT) were analyzed. These trials encompassed the following:

- the combination of indacaterol (IND) with mometasone furoate (MF) and glycopyrronium bromide (GB), marketed under the trade name Enerzair ^®^, and
- the combination of beclomethasone dipropionate (BDP) with formoterol fumarate (FF) and glycopyrronium bromide (GB), delivered via the pMDI HFA Modulite ^®^ inhaler, commercialized as Trimbow ^®^.

Drawing upon these data and the conclusions drawn from prior expert positions, the utilization of ICS/LABA/LAMA was recommended for optimal therapeutic outcomes in the following clinical scenarios:

◦ the coexistence of asthma and chronic obstructive pulmonary disease (COPD),
◦ lack of asthma control, i.e., the presence of disease symptoms despite treatment with high doses of ICS and LABA,
◦ frequent infectious exacerbations of the disease,
◦ rapid progressive decline in lung function parameters.

In the summary of the position it was stated:

“The growing body of evidence underscores the efficacy of triple therapy in patients who fail to attain adequate symptom control despite high-dose ICS therapy combined with LABA, thereby substantiating the rationale for therapeutic escalation from Step 4 to 5 as per GINA guidelines through the inclusion of LAMA, rather than more escalation of ICS dosage to high levels. This is particularly relevant for individuals who stand to benefit from dual bronchodilator therapy. Furthermore, it merits consideration to incorporate LAMA into the treatment regimen of patients managed at Step 4 according to GINA guidelines, wherein significant declines in spirometric parameters persist despite adherence to such therapeutic interventions”

[10].

The clinical efficacy of single inhaler triple therapies in asthma has undergone assessment by the European Medicines Agency (EMA) as part of the medicinal product registration process.

**Figure 1:**
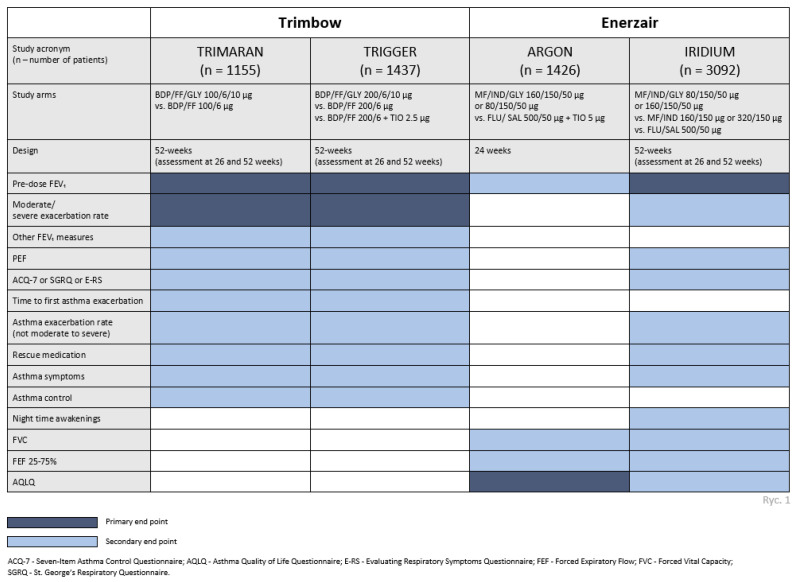
Clinical programs, phase III trials. A comparison of primary and secondary endpoints of two triple therapies currently registered in Europe for the treatment of asthma.

The EMA expects responsible parties (pharmaceutical companies) to provide evidence of the clinical efficacy of active substances, recommending studies targeting clinically relevant endpoints (Figure 1).

The recommendations stipulate the necessity for conducting a minimum of two clinical trials, wherein the primary endpoints entail the assessment of pre-dose forced expiratory volume in one second (FEV_1_) alterations, and the incidence rate of moderate to severe exacerbations of the disease.

Subsequent to the aforementioned clinical trials, the results of the efficacy of the evaluated therapies were submitted to the European Medicines Agency for registration of these products for the treatment of asthma patients (Table 1).

The EMA has granted registration to both formulations for the treatment of asthma in patients presenting with the following clinical conditions:

### 2.1. Trimbow (BDP/FF/GB):

“Maintenance treatment of asthma in patients with inadequate control of disease symptoms after treatment with a combination product of a long-acting beta-2 receptor agonist and a medium-dose inhaled glucocorticosteroid who have had one or more asthma exacerbations in the previous year.”

[11].

### 2.2. Enerzair (MF/IND/GB):

“Enerzair Breezhaler is indicated for the maintenance treatment of asthma in adult patients who have not achieved adequate disease control with maintenance combination therapy of a long-acting beta-2 agonist and a high-dose inhaled glucocorticosteroid, who have had at least one asthma exacerbation in the previous year.”

[12].

## 3. New Data: June 2021—March 2024

In addition to recommendations for asthma treatment derived from results of RCTs, the medical community anticipates further novel data post-registration of medicinal products. These commonly encompass results from the following:

- consecutive RCTs demonstrating clinical efficacy in a controlled setting in specific patient subgroups,
- experimental research employing novel imaging methods,
- post hoc analyses derived from RCTs targeting relevant features from the perspective of treatment response,
- data demonstrating the effectiveness of medications in real-world clinical practice, typically obtained through observational, non-interventional studies (such as real-life studies or medical registry data), conducted by healthcare practitioners within real patient settings.

The subsequent overview encapsulates pertinent literature items serving as the foundation upon which, according to expert opinions, new recommendations can be formulated and presented, augmenting the existing position:

### 3.1. BDP/FF/GB New Data

#### 3.1.1. Pulmonary Deposition

In a subsequent study aimed at assessing the in vivo lung deposition of radio-labeled technetium-99m BDP/FF/GB administrated via a pressurized metered-dose inhaler (pMDI), and the distribution of deposited medication within central (C) and peripheral (P) lung regions, gamma scintigraphy was employed. The study encompassed ten healthy volunteers and nine asthma patients. Secondary endpoints included the central to peripheral deposition ratio (C/P) and the normalized ratio of central to peripheral (sC/P) deposition.

Among asthma patients, the mean pulmonary deposition was recorded at 25.50%, whereas in healthy volunteers it stood at 22.74%. Approximately half of the administered dose localized in peripheral lung regions, constituting 0.52 ± 0.07 in healthy volunteers and 0.49 ± 0.06 in asthma patients, resulting in C/P ratios of 0.94 ± 0.25 and 1.06 ± 0.25, respectively. BDP/FF/GB demonstrated favorable tolerability.

The study’s findings affirmed that the extrafine particles delivered by BDP/FF/GB pMDI predominantly deposit in peripheral lung areas, with a comparable proportion of particles deposited in both central and peripheral regions. Crucially, the deposition patterns remained consistent between healthy volunteers and asthma patients, suggesting that clinical status does not exert a significant influence on pulmonary medication deposition.

#### 3.1.2. Pharmacokinetic Profile

Treatment guidelines currently do not distinguish between adults and adolescents (aged 12–17 years), concerning maintenance therapy options for asthma. While triple therapy is not presently approved for asthma treatment in this age group, there have been “off-label” treatment trials conducted for the adolescent population. In one such study, the pharmacokinetic/pharmacodynamic (PK/PD) profile for therapeutic and supra-therapeutic doses of BDP/FF/GB was compared between adults and adolescents. This comparison aimed to assess systemic exposure in adolescents relative to adults, demonstrating a similar safety profile across both age groups.

The study [13] findings revealed that systemic exposure following administration of BDP/FF/GB 400/24/50 μg via pMDI in adolescents did not surpass that observed in adults with controlled asthma. Furthermore, there were no significant discrepancies in terms of pharmacokinetic and pharmacodynamic parameters between the two age groups, and BDP/FF/GB exhibited good tolerability in both cohorts. In summary, there are no safety signals warranting a reduction in the dose of BDP/FF/GB in adolescents with asthma. However, additional studies, particularly with multiple doses, are warranted to assess the efficacy of BDP/FF/GB in adolescents with asthma. Fixed-dose inhaled triple therapy for severe asthma boasts a favorable safety profile, yet several potential drawbacks necessitate further investigation. 

#### 3.1.3. Normalization of Airflow Limitation

In asthma, persistent airflow limitation (PAL) is associated with poorer disease control, declining lung function, and increased risk of exacerbations. Through post hoc analyses [14], the relationship between PAL magnitude, assessed post-salbutamol administration during screening for the TRIGGER and TRIMARAN clinical trials, airflow limitation (AL) during extrafine-particle BDP/FF/GB vs. BDP/FF use, and the incidence of moderate/severe asthma exacerbations was examined. Asthmatic patients exhibiting PAL [15] tend to represent long-standing asthmatic cases, with a higher prevalence among males and smokers. Patients with PAL demonstrate more severe small airway dysfunction, manifested by increased conducting airway and lung parenchymal ventilation heterogeneity. Moreover, they exhibit distinctive inflammation patterns, characterized by elevated eosinophil levels in sputum and blood, increased blood monocyte counts, and decreased sputum macrophage percentages. PAL presence correlates with elevated asthma exacerbation risk that is independent of age, gender, smoking, eosinophilic inflammation severity, and disease severity.

Patients were categorized into the following subgroups based on PAL status during screening and AL throughout treatment:

- PAL presence at screening—defined as the FEV_1_/FVC ratio <0.7 at 10–15 min. post-salbutamol administration.
- AL during treatment—defined as the FEV_1_/FVC ratio <0.7 three hours post-medication administration during all post-randomization visits.

The majority of patients with PAL post-salbutamol administration at screening exhibited AL during all treatment visits (TRIMARAN—62.8%; TRIGGER—66.8%). Significantly more patients receiving extrafine-particle BDP/FF/GB achieved normalized airflow at screening compared to those on BDP/FF (TRIMARAN 44.1 vs. 33.1% [*p* = 0.003]; TRIGGER 40.1 vs. 26.0% [*p* < 0.001]). In patients with PAL (after salbutamol administration) at screening and subsequent AL normalization, exacerbation frequency was 15% (*p* = 0.105) and 19% (*p* = 0.039) lower in the TRIMARAN and TRIGGER groups compared to individuals with consistent AL.

A trend toward reduced exacerbations was observed in patients receiving BDP/FF/GB vs. BDP/FF, particularly in those achieving AL normalization.

Consequently, extrafine-particle BDP/FF/GB exhibited consistently superior efficacy compared to BDP/FF in patients with more reversible airway obstruction across all exacerbation analyses.

#### 3.1.4. Special Patient Subgroup—Stratification Based on Eosinophil Count

In post hoc analyses [16] stratifying patient subgroups based on blood eosinophil count, the TRIMARAN study revealed a more pronounced effect of BDP/FF/GB compared to BDP/FF on lung function parameters among individuals with ≤300 cells/μL. Conversely, in the TRIGGER study, the blood eosinophil count did not significantly influence the relative efficacy of BDP/FF/GB vs. BDP/FF.

#### 3.1.5. “Real Life” Studies

The evaluation of single inhaler triple therapy (SITT) on asthma control and health-related quality of life (HRQoL) in real-world settings remains incomplete.

The TriMaximize study [17], currently underway across several European countries, aims to report on the characteristics, treatment pathways, and health outcomes of 2600 patients diagnosed with moderate to severe asthma who were prescribed SITT—extrafine-particle BDP/FF/GB—in real-world settings. This multicenter, multinational, prospective, non-interventional study aims to gauge the impact of SITT on patients with moderate to severe asthma in a real-world practice. Demographic and clinical data are collected from patients’ medical records over a period spanning 12–36 months post-medication initiation. Changes in asthma control and HRQoL are assessed using the Asthma Control Test (ACT) and the Mini Asthma Quality of Life Questionnaire (Mini-AQLQ).

The results of a preliminary analysis of changes in the asthma control and HRQoL in the first 582 patients from Germany, the UK, Austria, and Denmark after 3 months of SITT treatment are presented. Upon study commencement, 77% of patients were receiving non-extrafine ICS/LABA treatment, while 23% were on non-extrafine ICS/LABA/LAMA therapy.

The mean ACT score in the general population at baseline stood at 14.4 points. Following 3 months of SITT treatment, the mean change in the ACT score from baseline was 3.3 points (*p* < 0.0001) in the general population and 3.7 points (*p* < 0.0001) in the SITT-treated population in patients previously managed with ICS/LABA, both surpassing the minimum clinically important difference (MCID) of 3 points. Likewise, the mean Mini-AQLQ score in the entire population at the study’s onset was 4.1 points. After 3 months of SITT therapy, the mean change in the Mini-AQLQ score from baseline was 0.7 points (*p* < 0.0001) in the overall population and 0.9 points (*p* < 0.0001) in patients previously treated with ICS/LABA, both surpassing the MCID value of 0.5 points. Over half (55.8%) of patients achieved the MCID for the Mini-AQLQ, signifying a favorable treatment response. Notably, in the subgroup of patients exhibiting a treatment response in Mini-AQLQ, a mean change in 5.8 points (*p* < 0.0001) in the ACT score from baseline was observed. 

This study represents a pioneering real-world investigation showcasing enhancements in quality of life and asthma control among individuals with uncontrolled moderate to severe asthma following 3 months of SITT treatment (extrafine BDP/FF/GB), subsequent to a switch from ICS/LABA or ICS/LABA/LAMA regimens. 

#### 3.1.6. Biological Therapy

An important aspect of asthma management entails determining whether triple therapy via a single inhaler can defer or obviate the necessity for biological agents in severe asthma cases. In a prospective study [18], 17 patients diagnosed with severe asthma, despite receiving high doses of ICS and additional controller medications (LABA, LAMA, LTRA) without achieving adequate symptom control—typical candidates for biologic therapy—were monitored. All patients transitioned to BDP/FF/GB (172/5/9 μg) 2 × 2 inhalations per day. Following 3 months of therapy, a noteworthy enhancement in the asthma control (ACT) score was observed (T0 vs. T3: 14.5 ± 5.6 vs. 19.0 ± 5.3, *p* < 0.01), alongside a reduction in the residual volume to total lung capacity (RV/TLC) ratio (T0 vs. T3: 53.1 ± 13.8 vs. 42.8 ± 16.1, *p* < 0.05). This decline in the RV/TLC ratio correlated with improved ventilation, gas exchange, and diminished symptom severity. Consequently, none of the participants necessitated biological therapy initiation. Preliminary findings suggest that 3 months of therapy with extrafine BDP/FF/GB from a single inhaler can significantly alleviate asthma symptoms and lung parenchyma hyperinflation, thus enabling the postponement of biological therapy introduction.

Biological therapies have significantly raised our expectations for asthma outcomes, introducing the following challenging new treatment goal: achieving clinical remission. However, attaining such clinical remission can often entail considerable expenses, encompassing manufacturing, administration, monitoring, and potential management of adverse effects. By deferring the onset of biological treatment through efficacious management of uncontrolled asthma via triple therapy from a single inhaler, both patients and healthcare systems may potentially alleviate financial burdens.

#### 3.1.7. Cost-Effectiveness

The cost-effectiveness of triple therapy utilizing BDP/FF/GB was evaluated across various scenarios [19]. In each scenario, medium and high doses were deemed cost-effective in comparison to equivalent doses of BDP/FF in adults experiencing uncontrolled asthma while using ICS/LABA, employing a standard willingness-to-pay threshold of 20,000 GBP/QALY from the perspective of the British NHS. Moreover, in all scenarios, high-dose BDP/FF/GB therapy emerged as the dominant strategy over high doses of BDP/FF combined with tiotropium in these patients.

#### 3.1.8. Severe Adverse Events (SAE)

Triple therapy from a single inhaler (combining ICS/LABA/LAMA) currently stands recommended as a controller option in Step 4 of asthma management, and as the preferred treatment in Step 5. However, thus far, no studies have systematically examined the potential drawbacks of this therapeutic approach in a large asthma patient population. Therefore, the purpose of the following study [20] was to quantify the potential drawbacks of fixed-dose combination (FDC) triple therapy in asthma. 

Data encompassing 7204 patients with asthma were obtained from the CAPTAIN, IRIDIUM, TRIMARAN, and TRIGGER trials. Comparative analyses between triple therapies and ICS/LABA regimens revealed no elevated risk of total severe adverse events (SAEs) (RR-0.99, 95% CI 0.83–1.18) and cardiac SAEs (RR-0.74, 95% CI 0.39–1.40). An increased risk of vascular SAEs was noted in the sensitivity analysis. However, the dose of ICS did not affect the risk of pneumonia. Ultimately, it was concluded that triple therapy represents a safe pharmacological treatment modality in patients with severe asthma, characterized by a favorable safety profile.

### 3.2. MF/IND/GB New Data

#### 3.2.1. Cost-Effectiveness

The cost-effectiveness of the IND/GB/MF combination [21] in a fixed dose was evaluated in comparison to multiple inhaler triple therapy (MITT), comprising salmeterol/fluticasone (SAL/FLU) and tiotropium (TIO), salmeterol/fluticasone, or IND/MF within adult asthma patients, from the perspective of the Italian healthcare system. The analysis revealed that IND/GB/MF treatment, measured by QALYs, led to a 3-month longer improvement in health-related quality of life compared to MITT treatment involving salmeterol/fluticasone and tiotropium. Additionally, IND/GB/MF treatment demonstrated cost-effectiveness relative to the comparators considered, within a cohort representative of adult asthma patients in Italy. In a separate Canadian study, estimated through cost-effectiveness analysis, a higher willingness-to-pay of 50,000 Canadian dollars/QALY was demonstrated for IND/GB/MF in patients with uncontrolled moderate to severe asthma, compared to SAL/FLU+TIO and SAL/FLU. 

#### 3.2.2. Safety

Data from four randomized trials [22], including the 52-week PALLADIUM (n = 2216) and IRIDIUM (n = 3092) trials, the 24-week ARGON trial (n = 1426), and the 12-week QUARTZ trial (n = 802), were analyzed to assess the cardiovascular safety of two novel inhaled fixed-dose combinations for asthma treatment, which are as follows: (i) ICS/LABA, mometasone furoate/indacaterol acetate (MF/IND); (ii) ICS/LABA/LAMA, MF/IND/glycopyrronium bromide (GB).

In summary, no evidence indicative of an elevated cardiovascular risk was identified upon the addition of IND to MF or the addition of GB to MF/IND. Similarly, no evidence suggested an increased cardiovascular risk with an escalated ICS dosage, or in comparison to SAL/FLU ± TIO. These findings align with the current understanding of the cardiovascular safety profile associated with the ICS/LABA and ICS/LABA/LAMA medication classes.

#### 3.2.3. Electronic Sensor with App

The registration and approval process of IND/GB/MF in the European Union featured the following innovative addition: an optional electronic sensor coupled with a smartphone app or analogous device, rendering it the inaugural “digital assistant” that can be prescribed alongside an asthma medication. Consequently, the European Medicines Agency acknowledged this registration as one of the “outstanding contributions to public health” within the domain of pneumonology/allergology, as outlined in its 2020 highlights report.

#### 3.2.4. Enhancement of Patient Adherence to Medical Recommendations

Suboptimal adherence to inhaled asthma therapy is associated with unsatisfactory clinical outcomes. Digital devices integrated with inhalers monitor medication adherence and issue reminders to patients in case of missed doses, thereby enhancing treatment outcomes for the disease. The referenced study [23] examined the effects of the IND/GB/MF Breezhaler, featuring an electronic sensor, on medication adherence and symptom management among asthma patients in real-world scenarios.

The retrospective analysis involved adult asthmatics who were prescribed Breezhaler with an electronic sensor. The evaluation encompassed several parameters, as follows: mean medication adherence, alterations in the asthma control test (ACT) score at baseline and 30 days post-evaluation, and the percentage of patients adhering to medication at a rate exceeding 80% (at days 16–30 and 76–90 of observation).

Among the 163 patients with data collected over the 90-day observation period, it was found that 82.8% and 72.4% of patients achieved at least 80% adherence to medical recommendations at 1 and 3 months, respectively.

Asthma control change was evaluated in 60% (n = 97) of patients who completed the observation. Initially, 33.0% of patients exhibited good control, which increased to 53.6% at the second ACT test after 30 days. Furthermore, the percentage of patients reporting very poor asthma control decreased from 43.3% at baseline to 22.7% at the second ACT assessment, indicating clinical improvement.

In their conclusions, the authors asserted that the utilization of the IND/GB/MF Breezhaler, equipped with a digital sensor, among asthma patients could potentially enhance symptom control and foster adherence to medical recommendations.

#### 3.2.5. Subgroup of Patients Stratified by Eosinophil Count

The baseline characteristics of a patient may potentially guide asthma treatment. The impact of the baseline eosinophil count on the effectiveness of MF/IND/GB in patients with inadequately controlled asthma was assessed through a post hoc analysis of the IRIDIUM study [24]. This analysis assessed the efficacy of MF/IND/GB in patient subgroups categorized by baseline eosinophil counts <300 and ≥300 cells/μL. The study encompassed a total of 3065 patients. Over a duration of 52 weeks, high-dose MF/IND/GB demonstrated reductions in the annual rates of moderate or severe asthma exacerbations by 23% and 10%, severe exacerbations by 31% and 15%, and all exacerbations by 33% and 10% compared to high-dose MF/IND for subgroups with <300 and ≥300 eosinocytes/μL, respectively. Furthermore, compared to high-dose FLU/SAL therapy, reductions of 33% and 41%, 45% and 42%, and 42% and 39% were observed, respectively.

The use of MF/IND/GB resulted in improvements in lung function and a reduction in the number of asthma exacerbations compared to MF/IND and FLU/SAL, irrespective of the baseline eosinophil count. This suggests that the efficacy of MF/IND/GB remained consistent across different levels of baseline eosinophil counts.

#### 3.2.6. Efficacy—Improvement in FEV_1_—Post Hoc Analysis of the IRIDIUM Study

In a post hoc analysis of the IRIDIUM study [25], the alteration in forced expiratory volume in one second (FEV_1_) following 26 weeks of treatment was assessed in patients who were administered medium doses of MF/IND/GB in comparison to those receiving high doses of MF/IND and FLU/SAL.

Medium-dose MF/IND/GB demonstrated an improvement in FEV_1_ compared to high-dose MF/IND (41 mL change; 95% CI 7–90) and high-dose FLU/SAL (88 mL change; 95% CI 39–137) at week 26. This favorable effect persisted until the 52nd week of the study. Exacerbation rates were 16% lower with medium doses of MF/IND/GB compared to high doses of MF/IND for all exacerbation cases (mild, moderate, and severe) and 21–30% lower compared to high doses of FLU/SAL for all (mild, moderate, and severe) exacerbations, moderate or severe exacerbations, and severe exacerbations over 52 weeks. No novel safety concerns were identified. In conclusion, the authors affirmed that medium-dose MF/IND/GB not only enhanced lung function, but also reduced asthma exacerbations compared to high-dose ICS/LABA, rendering it a promising therapeutic choice for patients in GINA 2022 Step 4.

#### 3.2.7. Efficacy—Patients with PAL Post Hoc Analysis of the IRIDIUM Study

A subsequent analysis of the IRIDIUM [25] trial results compiled data of patients with asthma divided into a subgroup with persistent airflow limitation (PAL) and a subgroup without PAL who received high-dose MF/IND/GB q.d. high-dose MF/IND q.d., or high-dose FLU/SAL b.i.d. Although the IRIDIUM study included patients with FEV_1_ <80% of predicted normal before bronchodilator administration, in this analysis, patients with PAL were characterized by FEV_1_ ≤80% of predicted normal and a FEV_1_/FVC ratio ≤0.7 after salbutamol administration. The authors concluded that continuous high-dose MF/IND/GB therapy comparably enhanced lung function parameters and decreased the annual frequency of asthma exacerbations across both analyzed subgroups, with and without PAL.

## 4. Conclusions

### 4.1. Position Statement 

Taking into account the data presented above, the experts uphold the previous recommendations outlined in the introduction to this update of the Position Statement.

The accumulated evidence suggests that adding LAMA to a medium- or high-dose ICS/LABA results in a decrease of asthma exacerbations compared to medium- or high-dose ICS/LABA alone, accompanied by sustained enhancements in lung function parameters.

SITT therapy has the potential to enhance asthma symptom management and postpone the requirement for initiating biologic therapy.

In real-life studies, transitioning from ICS/LABA or ICS/LABA/LAMA to extrafine-particle BDP/FF/GB treatment showcased an enhancement in quality of life and asthma control in patients with uncontrolled moderate to severe asthma. Additionally, adding LAMA to medium/high-dose ICS effectively enhances lung function parameters across various baseline characteristics, including gender, age, allergic status, disease duration, age of asthma onset, and airway obstruction severity.

Persistent airflow limitation (PAL) has emerged as a potential predictor of improved therapeutic response in asthma treatment. Notably, PAL is not confined to severe disease, but is also prevalent in a substantial proportion of patients with milder forms of the condition.

In patients with mild asthma, the presence of PAL is associated with the presence of eosinophilic inflammation and an increased risk of exacerbations. Post hoc analysis findings suggest that patients with milder asthma and PAL may benefit from increased treatment intensity considerations.

In studies comparing extrafine-particle BDP/FF/GB to BDP/FF, the efficacy of treatment was notably higher in patients exhibiting more reversible airway obstruction compared to those with less reversible airway obstruction.

Fixed-dose MF/IND/GB q.d. therapy demonstrated improved lung function parameters and a reduction in the annual number of asthma exacerbations in patient subgroups with and without PAL.

Adding LAMA to ICS/LABA does not compromise the good safety profile of these medications, as triple therapy with extrafine-particle BDP/FF/GB has shown a favorable safety profile.

Currently, there are no signals justifying the need to reduce doses of BDP/FF/GB for adolescents with asthma, although the medication is not registered for use in patients aged 12–17 years.

Extrafine-particle BDP/FF/GB pMDI therapy facilitates deep penetration of medication particles throughout the airways, depositing them in similar proportions in both central and peripheral lung regions. This deposition pattern remains consistent across healthy volunteers and asthma patients, suggesting that clinical status does not affect lung medication deposition.

Post hoc analyses stratified by blood eosinophil counts revealed a greater efficacy of BDP/FF/GB compared to BDP/FF in patients with ≤300 eosinophils/μL. However, the eosinophil count did not significantly impact the relative efficacy of BDP/FF/GB compared to BDP/FF in the TRIGGER study. Similarly, MF/IND/GB showed improved lung function and reduced asthma exacerbations compared to MF/IND and FLU/SAL, regardless of the baseline eosinophil count, warranting further prospective studies in this area.

Inadequate adherence to inhaled asthma therapy can compromise disease control. The development of inhalers equipped with digital sensors, such as the IND/GB/MF Breezhaler, improves treatment effectiveness by monitoring medication-use and sending reminders to patients if inhalations are missed.

SITT therapy, which combines three medications in a single inhaler, significantly improves patient adherence by reducing the number of inhalers needed, simplifying dosing regimens, and enhancing treatment flexibility and individualization, eliminating the need to master inhalation techniques from multiple inhalation systems, as well as by allowing for treatment flexibility and individualization through the use of low/medium doses of ICS and simpler dosing regimens.

Triple inhalation therapy from a single inhaler has been deemed cost-effective compared to triple therapy from multiple inhalers.

### 4.2. We Propose the Following

**Figure 2:**
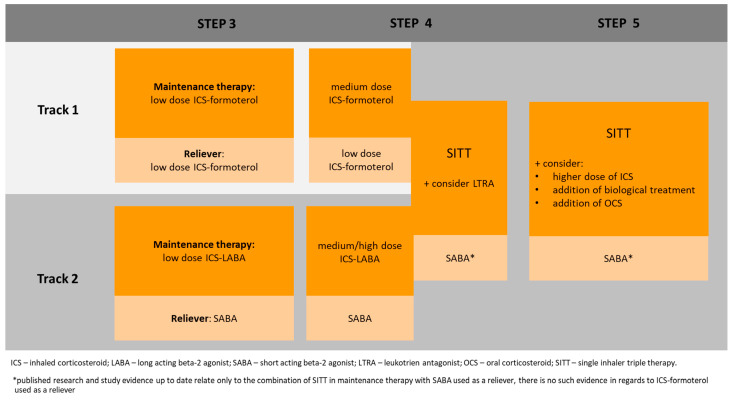
Polish Society of Allergology and the Polish Respiratory Society expert statement prepared based on the modified version of the GINA 2023 recommendations.

In adult patients experiencing suboptimal asthma control despite medium/high-dose ICS/LABA treatment—regardless of adherence to GINA-recommended strategies, such as MART therapy as a first-line approach or alternative second-line strategies—the preferred course for intensifying asthma therapy involves the addition of a LAMA, ideally in the form of SITT (Figure 2).

This means that we prefer SITT therapy over other alternative methods for intensifying asthma therapy, including the following:

- increasing the dose of ICS,
- adding LTRA,
- adding SAMA,
- initiating oral steroids,
- biological treatment.

## Figures and Tables

**Table 1:** Summary of the efficacy of SITT compared to ICS/LABA in phase III RCTs in patients with poorly controlled asthma [11,12,13].

Study	Improvement in FEV1 for SITT Compared to ICS/LABA	Reduction in Moderate/Severe Exacerbations with SITT Compared to ICS/LABA
TRIMARAN BDP/FF/GB vs. BDP/FF	57 mL (95% CI 15–99; *p* = 0.0080) for MD	15% (RR 0.85, 95% CI 0.73–0.99; *p* = 0.033) for MD
TRIGGER BDP/FF/GB vs. BDP/FF BDP/FF/GB vs. BDP/FF+TIO	73 mL (95% CI 26–120; *p* = 0.0025) for HD −45 mL [95% CI-103 to 13; *p* = 0.13] for HD	12% (RR 0.88, 95% CI 0.75–1.03; *p* = 0.11) for HD 7% (RR1-07, 95% CI 0-88–1-30; *p* = 0.50) for HD
IRIDIUM MF/IND/GB vs. MF/IND MF/IND/GB vs. FP/SLM	76 mL (*p* < 0.001) for MD 65 mL (*p* < 0.001) for HD 99 mL (*p*<0.001) for MD 119 mL (*p* < 0.001) for HD	13% (RR 0.87, 95% CI 0.71–1.06; *p* = 0.17) for MD 15% (RR 0.85, 95% CI 0.68–1.04; *p* = 0.12) for HD 19% r (RR 0.81, 95% CI 0.66–0.99; *p* = 0.041) for MD 36% (RR 0.64, 95% CI 0.52–0.78; *p* < 0.001) for HD
ARGON MF/IND/GB vs. FP/SLM+TIO	HD and MD of MF/IND/GLY were non-inferior to HD of FP/SLM+TIO for AQLQ (difference: 0.073 and −0.038, respectively; both *p* < 0.001) for HD of MF/IND/GLY improvement in trough FEV_1_ at week 8 (A: 67 mL; *p* = 0.007) at week 16 (A: 66 mL; *p* = 0.007) at week 24 (A: 96 mL; *p* < 0.001) vs. HD of FP/SLM+TIO and for MD of MF/IND/GLY at week 8 (A: 3 mL; *p* = 0.892) at week 16 (A: −2 mL; *p* = 0.945) at week 24 (A: 9 mL; *p* = 0.713) vs. HD of FP/SLM+TIO	for MD of MF/IND/GLY vs. HD of FP/SLM+TIO 4% increase (RR 1.04, 95% CI 0.77, 1.39; *p* = 0.798) for HD of MF/IND/GLY vs. HD of FP/SLM+TIO 12% reduction (RR 0.88, 95% CI 0.65, 1.19; *p* = 0.414)

HD—high dose, MD—medium dose, BDP—beclomathasone dipropionate, FP—fluticasone propionate; FEV—forced expiratory volume in the first secondd; FF—formoterol fumarate; GB—glycopyrronium bromide; ICS—inhaled corticosteroids; IND—indacaterol; LABA—long-acting ẞ2-adrenoceptor agonist; MF—mometasone furoate; RCT—randomized controlled trial; SITT—single-inhaler device/triple therapy from a single inhaler; SLM—salmeterol; TIO—tiotropium.

## Data Availability

The original contributions presented in the study are included in the article, further inquiries can be directed to the corresponding author.

## References

[1] National Health Fund Healthy Data. Report. https://ezdrowie.gov.pl/portal/home/badania-i-dane/zdrowe-dane/raporty/nfz-o-zdrowiu-astma.

[2] Claxton A.J., Cramer J., Pierce C. (2001). A systematic review of the associations between dose regimens and medication compliance. Clin. Ther..

[3] Lindsay J.T., Heaney L.G. (2013). Non adherence in difficult asthma—Facts, myths, and a time to act. Patient Prefer. Adherence.

[4] Elliott R.A. (2006). Poor adherence to anti-inflammatory medication in asthma. Dis. Manag. Health Out..

[5] Azzi E., Srour P., Armour C., Rand C., Bosnic-Anticevich S. (2017). Practice makes perfect: Self-reported adherence a positive marker of inhaler technique maintenance. NPJ Prim. Care. Respir. Med..

[6] Kuziemski K., Chazan R., Doboszyńska A., Domagała-Kulawik J., Jassem E., Porzezinska M., Kuna P., Kędziora K., Ziora D. (2018). Triple-drug therapy for chronic obstructive pulmonary disease from a single inhaler versus adherence to therapy. Adv. Respir. Med..

[7] GINA 2023 Report. https://ginasthma.org/2023-gina-main-report/.

[8] Śliwiński P. New Treatment Strategy for Asthma Patients. https://www.medexpress.pl/leki-technologie-medyczne/nowa-strategia-leczenia-dla-pacjentow-z-astma-79933/.

[9] Śliwiński P., Antczak A., Barczyk A., Jahnz-Różyk K., Kulus M., Kuna P., Kupczyk M. (2020). Expert position of the Polish Society of Allergology and the Polish Respiratory Society on new combination inhaled IND/MF and IND/GB/MF drugs for the treatment of asthma. Pneum. Pol..

[10] Śliwiński P., Antczak A., Barczyk A., Białas A.J., Jahnz-Różyk K., Kulus M., Kuna P., Kupczyk M. (2021). Expert position of the Polish Society of Allergology and the Polish Respiratory Society on new combination inhaled drugs for the treatment of asthma. Pneum. Pol..

[11] European Medicines Agency, TRIMBOW Summary of Product Chartacteristics. https://www.ema.europa.eu/en/documents/product-information/trimbow-epar-product-information_en.pdf.

[12] European Medicines Agency, ENERZAIR Summary of Product Characteristics. https://www.ema.europa.eu/en/documents/product-information/enerzair-breezhaler-epar-product-information_en.pdf.

[13] Kuna P., Jerzynska J., Martini M., Vele A., Barneschi I., Mariotti F., Georges G., Ciurlia G. (2022). Pharmacokinetics of extrafine beclomethasone dipropionate/formoterol fumarate/glycopyrronium bromide in adolescent and adult patients with asthma. Pharmacol. Res. Perspect..

[14] Papi A., Singh D., Virchow J.C., Canonica G.W., Vele A., Georges G. (2022). Normalization of airflow limitation in asthma: Post-hoc analyses of TRIMARAN and TRIGGER. Clin. Transl. Allergy..

[15] Kole T.M., Vanden Berghe E., Kraft M., Vonk J.M., Nawijn M.C., Siddiqui S., Sun K., Fabbri L.M., Rabe K.F., Chung K.F. (2023). Predictors and associations of the persistent airflow limitation phenotype in asthma: A post-hoc analysis of the ATLANTIS study. Lancet Respir. Med..

[16] Singh D., Virchow J.C., Canonica G.W., Vele A., Kots M., Georges G., Papi A. (2020). Determinants of response to inhaled extrafine triple therapy in asthma: Analyses of TRIMARAN and TRIGGER. Respir. Res..

[17] Gessner C., Akyildiz B., Pohl W., Nachtigall D., Russell R., Bogoevska V., Ulrik C.S., Watz H. (2023). Impact of extrafine formulation Single-inhaler Triple Therapy on asthma control and health-related quality of life after three months of treatment in patients with asthma: Trimaximize—A real-world view from Germany, United Kingdom, Austria and Denmark. Am. J. Respir. Crit. Care Med..

[18] Dragonieri S., Quaranta V.N., Portacci A., Carpagnano G.E. (2023). Can single-inhaler beclomethasone dipropionate/formoterol fumarate/glycopyrronium therapy postpone or save biologics for severe asthma?. Pulm. Pharmacol. Ther..

[19] Orlovic M., Magni T., Lukyanov V., Guerra I., Madoni A. (2022). Cost-effectiveness of single-inhaler extrafine beclomethasone dipropionate/formoterol fumarate/glycopyrronium in patients with uncontrolled asthma in England. Respir. Med..

[20] Rogliani P., Cavalli F., Chetta A., Cazzola M., Calzetta L. (2022). Potential drawbacks of ICS/LABA/LAMA triple fixed-dose combination therapy in the treatment of asthma: A quantitative synthesis of safety profile. J. Asthma. Allergy.

[21] Mangia P.P., Gallo O., Ritrovato D., Pradelli L. (2021). Cost-utility analysis of fixed-dose combination of indacaterol acetate, glycopyrronium bromide and mometasone furoate as a maintenance treatment in adult patients with asthma not adequately controlled with a maintenance combination of a long-acting beta2-agonist and a high dose of an inhaled corticosteroid who experienced one or more asthma exacerbations in the previous year. Clin. Drug. Investig..

[22] Scosyrev E., van Zyl-Smit R., Kerstjens H., Gessner C., Kornmann O., Jain D., Aubrun E., D’Andrea P., Hosoe M., Pethe A. (2021). Cardiovascular safety of mometasone/indacaterol and mometasone/indacaterol/glycopyrronium once-daily fixed-dose combinations in asthma: Pooled analysis of phase 3 trials. Respir. Med..

[23] Woehrle H., Mastoridis P., Stempel D., Kaye L., Vuong V., Mezzi K. (2023). Medication adherence and asthma control with once-daily indacaterol/glycopyrronium/mometasone furoate Breezhaler Digital Companion: 90-day analysis from Germany. Pulm. Ther..

[24] Van Zyl-Smit R.N., Kerstjens H.A.M., Maspero J., Tanase A.M., Lawrence D., Mezzi K., D’Andrea P., Chapman K.R. (2023). Triple therapy with mometasone/indacaterol/glycopyrronium or doubling the ICS/LABA dose in GINA Step 4: IRIDIUM Analyses. Pulm. Ther..

[25] Van Zyl-Smit R.N., Kerstjens H.A., Maspero J.F., Kostikas K., Hosoe M., Tanase A.M., D’Andrea P., Mezzi K., Brittain D., Lawrence D. (2023). Efficacy of once-daily, single-inhaler, fixed-dose combination of mometasone/indacaterol/glycopyrronium in patients with asthma with or without persistent airflow limitation: Post-hoc analysis from the IRIDIUM study. Respir. Med..

